# 

**Published:** 1888-01-14

**Authors:** 


CONTENTS.

**Medical Journalism** - - - - - - - - 261

**The Limits and Influences of a Nurse's Vocation.** By R. BRUDENELL CARTER, F.R.C.S. - - - - - 263

**A Working-Class View of Our Hospitals.** By the Chairman of Workmen's Governors, Sunderland Infirmary - 264

**Christmas at the Hospitals.**—Ophthalmic Hospital, Moorfields.—Seamen's Hospital, Greenwich.—Oldham Infirmary.—Clapton Hospital, Wakefield. By our Commissioners and Correspondents - - - - - - - - 265

**The Doctor's Pulpit.**—"A little Knowledge." - - - 266

**The Practitioner's Clinic.** By C. R. ILLINGWORTH, M.D., M.R.C.S.—Pyrexia and Anti-Pyretics - - - 267

**Notes and News** - - - - - - - 268

**Everybody's Page** - - - - - - - 270

**Annotations.**—The Women's Jubilee Offering.—The Origin of Genius - - - - - - - - 271

**Rachel Challice's Vow.** By LENA NEVILLE, Author of "A Future on Trust," etc. - - - - - - 272

**The Biology of the Future.** By W. E. FOTHERGILL, M.A. 274

**The Common Accidents of Every-day Life.** By ROBERT A. P. FORRESTER, B.A. Dub.—Childhood: Greenstick Fracture - - - - - - - - 275

**The Theatres** - - - - - - - - iv.

**Amusements with Profit for all Ages.**—For Men and Women.—"The Hospital" Work Competition.—Children's Corner, etc. - - - - - - - - iv.

**The average issue of "The Hospital" now exceeds 10,000 COPIES WEEKLY, and the circulation is steadily increasing week by week.**

